# Comparing swab- and different symptoms-based strategies to ascertain COVID-19 recovery in healthcare workers: a cost-effectiveness analysis

**DOI:** 10.1186/s12962-022-00385-w

**Published:** 2022-09-12

**Authors:** Roberto Benoni, Irene Campagna, Francesca Moretti, Stefano Tardivo

**Affiliations:** 1 Postgraduate School of Hygiene and Preventive Medicine, University of Verona, 37134 Verona, Italy; 2 Department of Diagnostics and Public Health, Section of Hygiene, University of Verona, Strada Le Grazie, 8, 37134 Verona, Italy

**Keywords:** Cost-effectiveness, COVID-19, Recovery time, Healthcare workers, Economic evaluation

## Abstract

**Objective:**

Given the human and economic cost of the COVID-19 pandemic, protecting healthcare workers (HCW) and ensuring continuity of care is critical. The aim of this study is to evaluate the cost-effectiveness of different strategies to ascertain COVID-19 recovery in HCWs.

**Methods:**

Data were collected from the hospital health surveillance program on HCWs at the University Hospital of Verona between 29/02/2020 and 14/04/2021. The diagnosis of SARS-CoV-2 infection and the assessment of the recovery were made through RT-PCR on oro-nasopharyngeal swab-sample. Recovery time and probability were estimated through Kaplan–Meier estimate. For each recovery assessment strategy costs (laboratory diagnostics and human resources), expressed in local currency (euro—€), and working days saved (WDS—effectiveness) were estimated. A decision-tree was created where each knot was a time point scheduled by the different recovery assessment strategies. A Monte Carlo simulation method was used, and probabilistic sensitivity analysis assessed the effect of input uncertainty.

**Results:**

In the study period 916 (9.9%) HCWs tested positive. Recovery time through symptom-based strategy (21 days 0.95 CI 16–24) was significantly lower compared to swab-based one (25 days 0.95 CI 23–28, p < 0.001). The swab-based strategy was dominated by all symptoms-based ones. Symptoms-based with a swab on days 14 and 17 had an ICER of 2 €/WDS and 27 €/WDS compared to the one scheduled on days 10 and 17 and with only one swab on the 17th day.

**Conclusions:**

Scheduling swabs on days 14 and 17 in a symptom-based strategy was the most cost-effective, saving 7.5 more working days than the standard one with swabs on days 10 and 17.

**Supplementary Information:**

The online version contains supplementary material available at 10.1186/s12962-022-00385-w.

## Background

Since the beginning of the coronavirus disease 2019 (COVID-19) pandemic more than 4,812,000 people in Italy have been infected by SARS-CoV-2 and more than 132,000 have died because of it [1]. Healthcare workers (HCWs), due to the nature of their profession, have been among the most affected by the pandemic, especially at its onset, when knowledge about its means of transmission and containment was scarce [2]. According to reports sent by member States to the World Health Organization (WHO) during the first wave of the pandemic (March–May 2020) HCWs experienced more than triple the risk of infection compared to that of the general population [3]. As of November 8, 2021, in Italy 146,691 HCWs have been infected [4]. The WHO estimated that between January 2020 and May 2021 in Italy 3970 HCWs may have died [3].

Premature mortality cost COVID-19-associated was estimated as €137,789 per excess death across Europe with Italy and Spain being the most burdened countries during the first wave [5]. Striking the right balance between public health measures to control the spread of infection and the broader needs of society and the economy should be a priority.

The dimensions of the phenomenon had unprecedented and costly consequences on the sustainability of the National Healthcare System [6]. Preserving the health of HCWs has been considered a priority since the onset of the pandemic. To this avail, the University Hospital of Verona, a main hub in the Veneto region in Italy’s Northeast, following national and regional guidelines [7] had set up a surveillance program in March 2020 [8]. Along with regular testing screening of all staff and close contacts’ surveillance, it entailed the execution of periodical swabs for HCWs who resulted positive in order to allow their as prompt as possible return to work. In October 2020 the program was revised according to National guidelines [9].

Following WHO assessments [10] a symptoms-based strategy was implemented, reducing the number of swab tests required to return to work. This happened before the beginning of the second wave of the pandemic, which in Italy occurred between October and December 2020 and was characterized by a much higher number of cases than the first wave and less restrictive containment measures. A study conducted in Switzerland demonstrated that a regular testing regime of HCWs is optimal to prevent transmission among coworkers while keeping work output high and economic cost low [11], but, to our knowledge, few data are available on the most cost-effective testing strategy of positive HCWs.

The aims of this study were to describe the infected HCWs characteristics and recovery time between the two waves of the pandemic and to evaluate the costs and effectiveness of different recovery assessment strategies.

## Methods

A retrospective study was conducted using data from health surveillance program (HSP) of the University Hospital of Verona (UHV) located in the Veneto Region (Italy). Data referred to the period between February 29, 2020 (date of the first swab collected in the UHV) and April 14, 2021.

### Health surveillance program

The HSP was established at a national level [7] and implemented in regional setting according to local procedures [12], as described in another study [13].

**Fig. 1:**
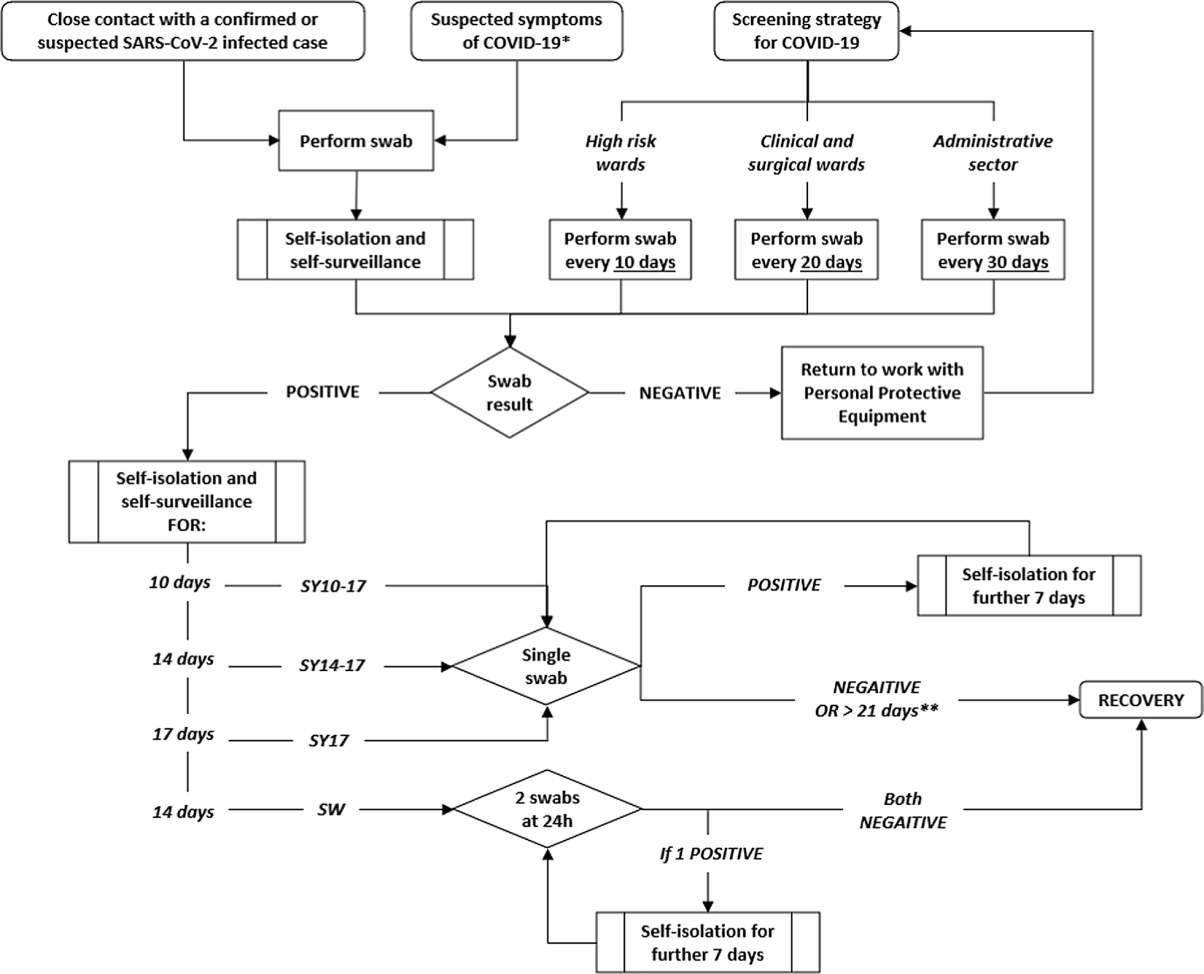
Flow Chart for Health Surveillance Program pathways and recovery assessment strategy involved in the study. SW: swab-based strategy; SY10-17: symptoms-based strategy with swabs scheduled at 10 and 17 days; SY14-17: symptoms-based strategy with swabs scheduled at 14 and 17 days; SY17: symptoms-based strategy with one swab at 17 days. *Fever or chills, cough, shortness of breath or difficulty breathing, fatigue, muscle or body aches, headache, new loss of taste or smell, sore throat, congestion or runny nose, nausea or vomiting, diarrhea [15]. **From first positive swab or symptoms onset (with at least 7 days symptoms-free)

The HSP provided the swab test based on three ways (Fig. 1): HCWs with COVID-19 related symptoms, HCWs with a close contact with a SARS-CoV-2 infected individual and through a screening program for all remaining HCWs. Close contact was defined as either a contact with a SARS-CoV-2 infected individual without any personal protective equipment, within two meters and for more than 15 min; furthermore, close contact could also be considered as an unprotected direct contact with secretions of a SARS-CoV-2 infected case. High-risk wards were screened every 10 days, all the others clinical and surgical wards every 20 days, whereas the employees of the administrative sector were tested every 30 days. In the UHV, Intensive Care Units, Infectious and Respiratory Diseases wards and COVID Units were considered at high-risk.

If an HCW tested positive to any of the swabs carried out by the HSP a home self-isolation was recommended for different periods depending on the recovery strategies adopted.

### Recovery assessment strategies

Swab-based strategy was used from February 28, 2020, until October 12, 2020. According to this, positive HCWs required to isolate for at least 14 days (or longer until symptoms resolution) and then to perform two swabs taken 24 h apart. Only if both swabs resulted negative, the HCW was considered recovered. In case one of the two swabs tested positive, the HCW had to repeat both swabs after 7 days [14].

From October 12, 2020, the Italian government adopted the symptoms-based strategy suggested by the World Health Organization to ascertain the COVID-19 recovery [9]. An infected asymptomatic individual was considered recovered if the RT-PCR swab test performed 10 days after the first positive one tested negative. In case of symptomatic patients, the swab had to be taken 10 days after the symptoms’ onset, after at least three days symptoms-free. If this first swab tested positive, it had to be repeated after 7 days until it proved negative or 21 days (with at least 7 days symptoms-free) since the first positive swab or onset of symptoms.

### Ethics

According to Law Decree N.14 of March 9, 2020, personal data were collected to guarantee public health and to ensure the diagnosis and the assistance of the infected subjects in the context of COVID-19 emergency [16]. Data were collected exclusively for the purpose of the HSP, it was anonymized and presented in an aggregated format as to ensure privacy of the subjects. This research was performed following the ethical standards of the 1964 Declaration of Helsinki and was launched and approved by the Institutional Board of the Veneto Regional Health Authority [17].

### Cost-effectiveness analysis (CEA)

#### Strategies included

The recovery assessment strategies included in the CEA were standard swab-based (*SW*) and symptoms-based (*SY10-17*) strategies. Kaplan–Meier estimate for right censored data was applied to explore the median recovery time. According to Kaplan–Meier recovery probability estimation, two different symptoms-based strategies with swabs scheduled at 14 and 17 days (SY14-17) and with only one swab at 17 days (SY17) were explored in this analysis. A total of four strategies were included in the analysis (Fig. 1).

#### Costs

All costs were collected and analysed as local currency (euro—€). The relevant costs included in the CEA were: the resources needed to provide the microbiological infection diagnosis and human resources workload per day (Table 1). Costs related to SARS-CoV-2 diagnosis were considered as the cost of the swabs and reagents for sample processing, cost of personal protective equipment and cost of maintenance. Cost of human resources was estimated as the cost of a half work shift (4 h), according to Italian national collective agreement [18], calculated for the staff implemented to get the diagnosis (i.e., 2 nurses to run the swab sampling clinic and 1 laboratory technicians for specimens’ analysis), divided by the mean number of HCWs needed to be tested per day.

**Table 1:** Mean cost with 0.95 confidential interval (CI) for gamma distribution estimated for the recovery assessment of a SARS-CoV-2 infected heath care workers

	Mean cost (€)	Variation (0.95 CI)	References
SARS-CoV-2 diagnosis			
SARS-CoV-2 Test Kit (unit)	14,15	(11.82, 16.63)	[19]
Maintenance costs	20.0	(16.0, 24.0)	[20]
PPE/pppd (KN95 + gloves)	2,62	(1.02, 5.39)	[19, 21]
Human resources			
Nurse (4 h)	36,51	(34.71, 38.20)	[18]
Laboratory technician (4 h)	31,88	(29.48, 34.31)	[18]

PPE: personal protective equipment: pppd: per person per day

#### Outcome (effectiveness)

Effectiveness was considered as the amount of working days saved (WDS) in a three-month period, starting form the first positive swab, thanks to the early reintroduction of HCW into his workplace through the different recovery assessment strategies.

#### Analysis

A decision tree was created. Every node was a time point scheduled by the four competing strategies with different probabilities of proved positive or negative estimated through the previous survival analysis (see Additional file 1: Fig. S1). Triangular distribution was applied for recovery probability (see Additional file 1: Table S1). Monte Carlo simulation, estimating the effect of parameters variability in recovery trajectory and individual responsiveness to recovery assessment strategies, was used with number of iterations set at 10,000 [22, 23]. Steps of the algorithm used in the Monte Carlo simulation were shown in the Additional file 1, while parameters variability for the recovery probability at every knot of the decision tree and the costs included in the analysis were reported in Additional file 1: Table S1 and Table 1, respectively. Costs were assumed with a gamma distribution. Number of WDS had a normal distribution.

Willingness to pay (WTP) was set at €160, estimating as the maximum cost of microbiological infection diagnosis and human resources per day per WDS paid by the hospital.

Data were analysed by comparing the four competing strategies reporting crude and incremental costs, incremental effectiveness, and efficiency indicators: Incremental cost-effectiveness ratio (ICER) and incremental net monetary benefit (INMB). Incremental measures were estimated with a 0.95 confidence interval. To consider the uncertainty of parameters, one-way sensitivity analysis (OWSA) and probabilistic sensitivity analysis (PSA) were performed. Results were showed as tornado diagram, cost-effectiveness scatterplot, ICER plot and acceptability curve of cost-effectiveness [24, 25].

### Statistical analysis

In the descriptive analysis, frequency rates and percentages were used for categorical variables and medians with interquartile range for continuous variables. Clopper Pearson method with an established 95% confidence interval was used for cumulative incidence of positive HCWs. Fisher’s exact test and χ^2^ test, as appropriate, and Mann–Whitney-U non-parametric test were used to compare categorical and continuous variables, respectively. The association between time of recovery, as dependent variable, and clinical and demographic characteristics was investigated via Cox proportional hazard regression. To evaluate the trend over time of recovery assessment costs in relation with the number of infected cases, a linear regression model was fitted. Regression coefficient beta was shown with standard error (SE). Interaction term was fitted between number of daily infected HCWs and the types of strategies as independent variables; dependent variable was the daily recovery assessment cost.

A p-value < 0.05 was considered significant. All analyses were performed using R software (version 4.1.1).

## RESULTS

### Infected healthcare workers characteristics

In the study period 9174 HCWs (male 2828—30.8%; female 6346—69.2%) were tested for SARS-CoV-2 and 916 (9.9%; 0.95 CI 9.4–10.6%) of these were positive. Positive rate was not significantly different between sexes (male n = 258—28.2%; female n = 658—71.8%; p = 0.072). SARS-CoV-2 infected HCWs had a median age of 46.1 years and were older than non-infected individuals (41.1 years; p < 0.001). Symptoms were reported by 508 HCWs (55.2%) with no differences based on sex (p = 0.987). Symptomatic HCWs had a median age of 47.3 years and were significantly older than asymptomatic ones (43.8; p = 0.003). Eighty-two HCWs (16.6%) developed symptoms after the execution of the positive swab test with a median time of 2 days (IQR1-3). In the overall period 18 (2.0%) of the SARS-CoV-2 infected HCWs needed hospitalization with a median age of 64.7 years and were significantly older compared to non-hospitalized ones (45.7 years; p = 0.005).

During the first phase (from February 2020 to October 2020) 8049 HCWs were tested and 255 (3.2%; 0.95 CI 3.0%-3.6%) resulted positive; during the second period (from October 2020 to April 2021) 8486 HCWs were tested and 661 (7.8%; 0.95 CI 7.2%-8.4%) were positive. No differences were found between infected HCWs during the first and second waves based on age (p = 0.526). There was a weak evidence of a difference based on sex (p = 0.056). Symptom’s development was not significantly different between the two periods (p = 0.240). Job position of the HCWs tested positive was not different between the two periods considered in the analysis (p = 0.086, Table S2). In the first period 12 HCWs (4.9%) needed hospitalization while 6 HCWs (0.9%) were hospitalized during the second one (p < 0.001).

### Recovery time

Median time of recovery, considering both strategies together, was 21.0 days (0.95 CI 20–21). Considering the swab- and symptom-based strategies, the median recovery time was 25 days (0.95 CI 23–28) and 21 days (0.95 CI 16–24), respectively. Considering the symptom-based strategy, 17% and 41% of HCWs tested negative at 10 and at 17 days, respectively, from the first positive swab or the symptoms’ onset.

In cox-regression model, symptoms-based strategy showed a significantly lower time to ascertain the COVID-19 recovery (p < 0.001, Table 2). HCWs with symptoms and those who needed hospitalization had a median recovery time of 21 (0.95 CI 21–21) and 36 days (0.95 CI 31–49), respectively, and it was significantly longer compared to asymptomatic HCWs and non-hospitalized ones (17 days, p < 0.001 and 21 days p = 0.017), regardless of the strategies used to ascertain the recovery.

**Table 2:** Kaplan Meier estimation of recovery time and recovery Hazard Ratios (HR) estimated in the multivariate Cox Proportional hazard model

	Kaplan–Meier estimates			Cox regression		
	N Events	Median (days)	0.95 CI	HR	0.95 CI	p-value
Overall	913	21	20–21			
Sex						
Male	257	21	20–21	0.99	0.85–1.14	0.862
Female	656	21	20–21	Reference		
Age (years)				1.00	0.99–1.00	0.514
22–29	116	18	17–21			
30–39	221	20	18–21			
40–49	203	21	19–21			
50–59	294	21	19–21			
60–67	79	21	20–21			
Recovery assessment strategy						
Swab-based	210	25	23–28	0.18	0.14–0.22	< 0.001
Symptoms-based	26	21	16–24	Reference		
Symptoms						
Yes	508	21	21–21	0.65	0.56–0.74	< 0.001
No	405	17	17–18	Reference		
Hospitalization						
Yes	18	36	31–49	0.15	0.09–0.26	0.017
No	895	21	20–21	Reference		

### Cost-effectiveness

**Fig. 2:**
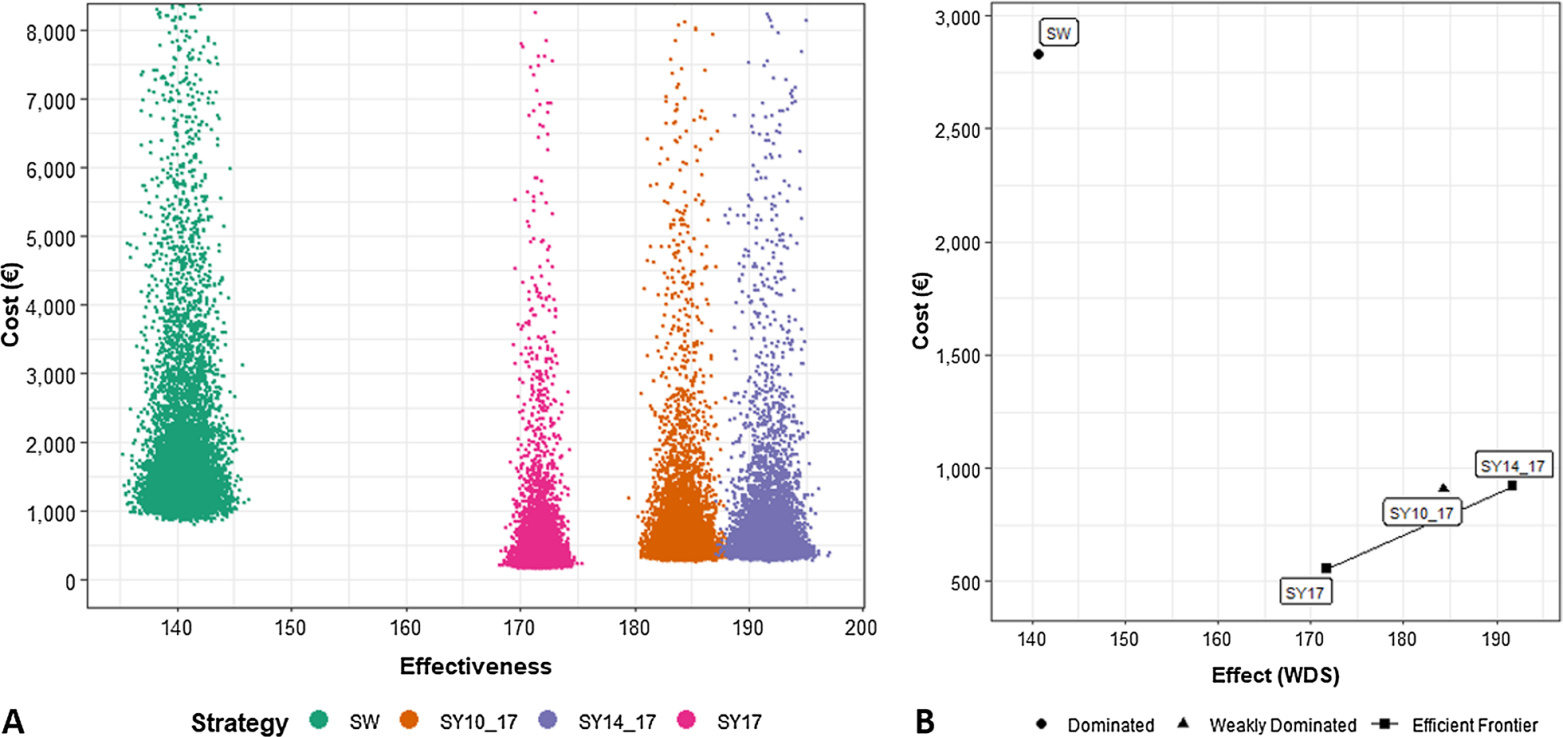
Scatterplot for 10,000 Monte Carlo iterations of costs and effectiveness for the four competing strategies (**A**). Incremental cost-effectiveness ratio plot. SW was dominated by all the different symptoms-based strategies. SY14-17 was the most cost-effectiveness strategy (**B**). SW: swab-based strategy; SY10-17: symptoms-based strategy with swabs scheduled at 10 and 17 days; SY14-17: symptoms-based strategy with swabs scheduled at 14 and 17 days; SY17: symptoms-based strategy with one swab at 17 days

The crude cost for the four competing strategies (SW, SY10-17, SY14-17, SY17) was €3688.9 (SD 48,496.9), €1180.9 (SD 15,413.1), €1198.8 (SD 15,690.8) and €722.8 (SD 9433.8), respectively. The mean number of WDS (effectiveness) was 140.7 (SD 1.5) for the SW strategy, 184.3 (SD 1.3), 191.7 (SD 1.4) and 171.7 SD (0.9) for the SY10-17, SY14-17 and SY17 strategies (Fig. 2A). SW strategy was dominated by all three symptoms-based strategies since had positive incremental cost and negative effectiveness (Fig. 2B). Incremental cost and effectiveness were shown in Table 3. Comparing the symptoms-based strategies, SY14-17 proved to be more cost-effective when compared to both SY10-17 and SY17 with an ICER of 2 €/WDS and 27 €/WDS and an INMB of €11,820 and €2730 respectively.

**Table 3:** Incremental (Δ) number of working days saved (WDS) thanks to the different recovery assessment strategies (effectiveness), incremental (Δ) cost, incremental cost-effectiveness ratio (ICER) and incremental net monetary benefit (INMB) between swab-based strategy (SW) and the three different symptoms-based and comparing the competing symptoms-based strategies with swabs scheduled at 10 and 17 days (SY10-17), 14 and 17 days (SY14-17) or only 17 days (SY17)

	Δ effectiveness (WDS)	Δ cost (€)	ICER (€/WDS)	INMB (€)
Strategies				
SW	Reference	Reference	Reference	Reference
SY10-17	43.6 (39.7; 47.5)	− 2508 (− 8100; − 666)	Dominates	9480 (7231; 15,349)
SY14-17	51.1 (47.1; 55.2)	− 2490 (− 8043; − 662	Dominates	10,662 (8408; 16,314)
SY17	31.0 (27.6; 34.6)	− 2966 (− 9589; − 789)	Dominates	7933 (5420; 14,647)
Strategies				
SY10-17	Reference	Reference	Reference	Reference
SY14-17	7.5 (3.8; 11.2)	18 (4; 55)	2	1182 (590; 1779)
SY17	− 12.5 (− 15.6; − 9.5)	− 458 (− 1484; − 122)	37	− 1547 (− 2306; − 473)
Strategies				
SY14-17	Reference	Reference	Reference	Reference
SY17	− 20.0 (− 23.3; − 16.8)	− 476 (− 1551; − 126)	24	− 2730 (− 3529; − 1612)

**Fig. 3:**
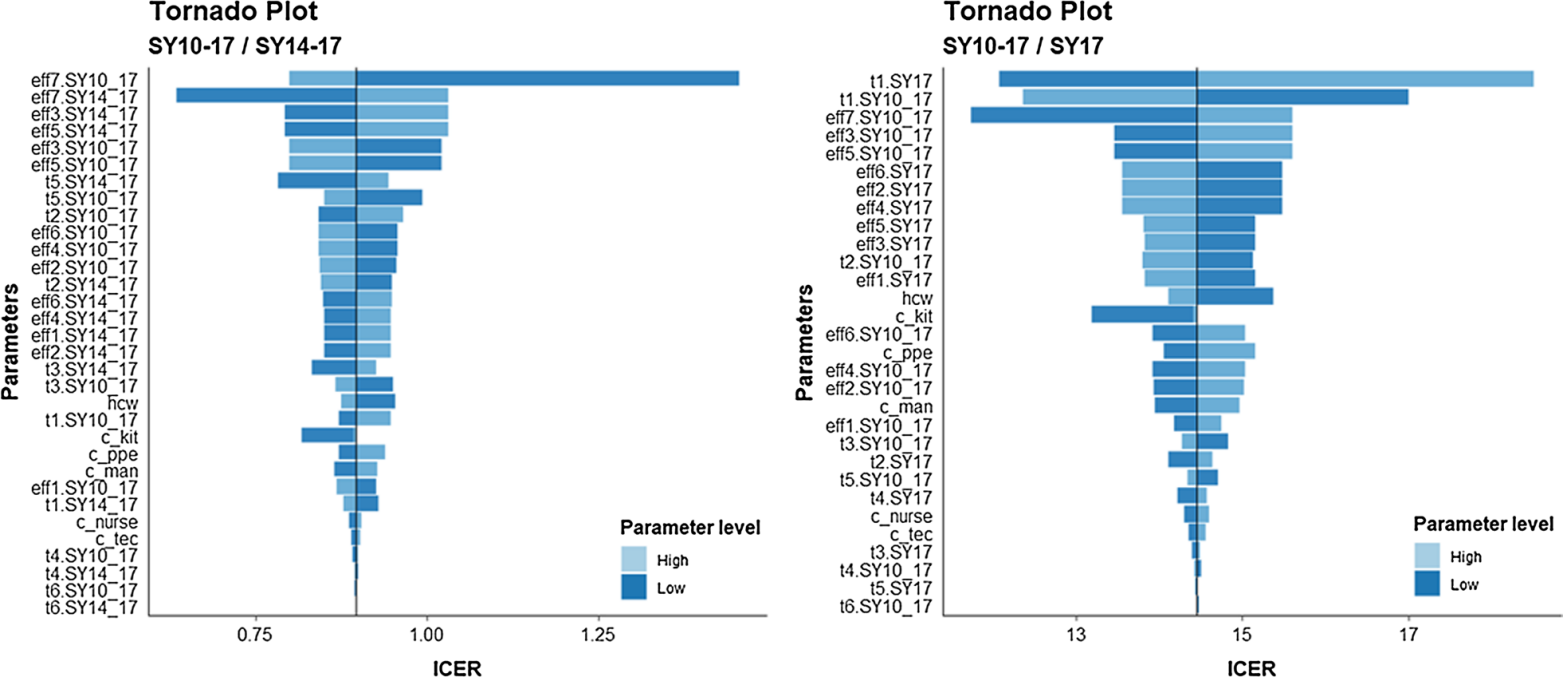
Tornado diagram of one-way sensitivity analyses comparing symptoms-based strategy with swabs scheduled at 10 and 17 days (SY10-17) with alternative symptoms-based strategy scheduled at 14 and 17 days (SY14-17, **A**) or only one swab at 17 days (SY17, **B**). t: recovery probability at every time points in the different strategies, eff: working days saved (effectiveness), the number after parameter “t” or “eff” refers to the time point of the decision tree (i.e. t1_SY17 refers to the recovery probability at the first time point in the symptoms based strategy owith nly one swab at 17 days), hcw: number of health care workers to be tested, c_kit: cost of SARS-CoV-2 test kit, c_ppe: cost of personal protective equipment, c_man: cost of maintenance, c_nurse and c_tec: cost oh human resources (nurse and laboratory technicians)

The tornado plot (Fig. 2) showed that estimate ICER was robust around parameters variability comparing SY14-17 and SY17 with SY10-17 strategy. The greatest impact on ICER was the variability in recovery probability at the first time point of SY10-17 and SY17 (Fig. 3 panel B).

**Fig. 4:**
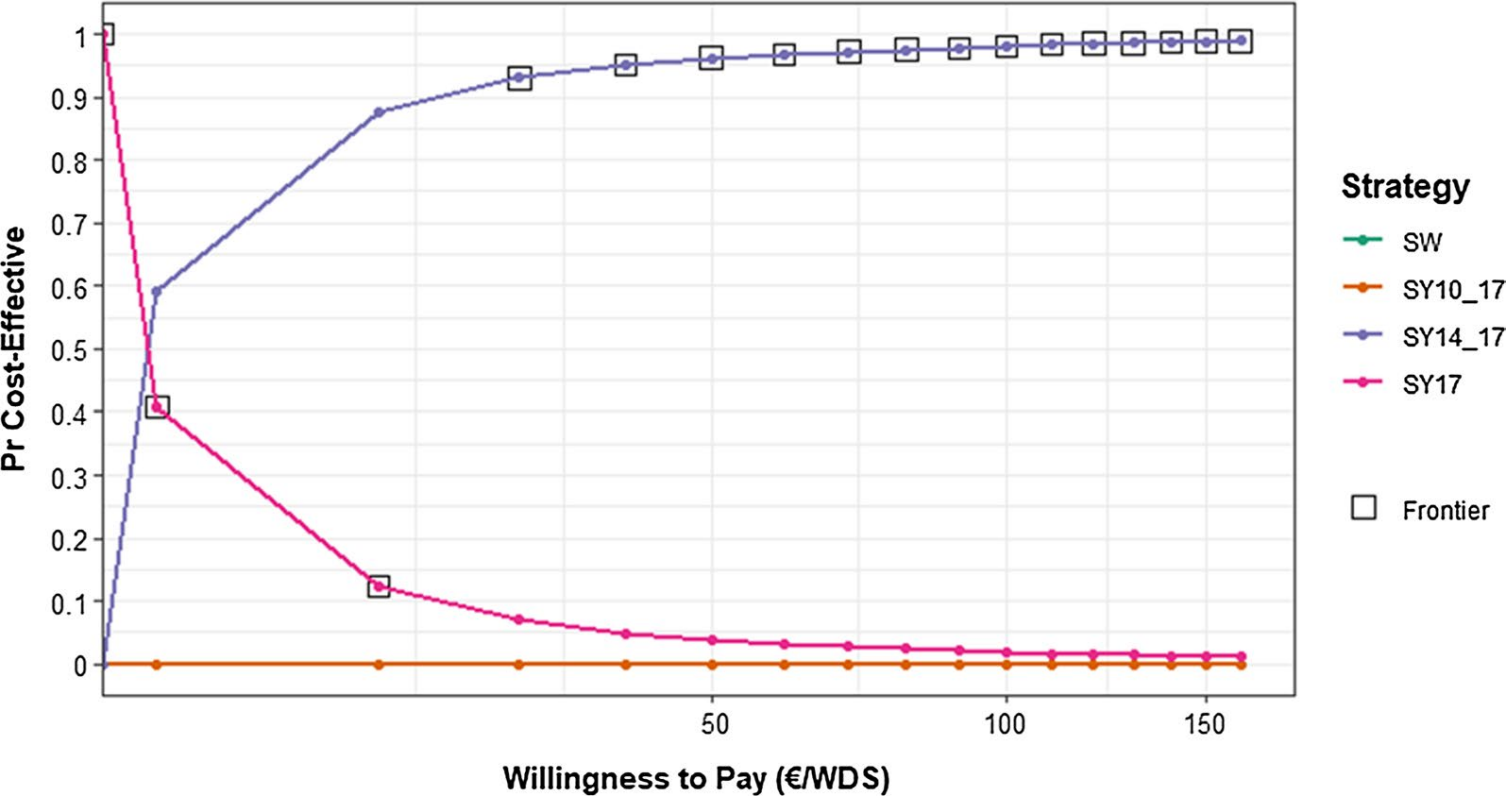
Cost-effectiveness Acceptability Curve of the four competing strategies: SW: swab-based strategy; SY10-17: symptoms-based strategy with swabs scheduled at 10 and 17 days; SY14-17: symptoms-based strategy with swabs scheduled at 14 and 17 days; SY17: symptoms-based strategy with one swab at 17 days. Willingness to pay (WTP) was set at 160€ with interval of 10€ and is expressed as €/WDS: working days saved

Acceptability curve demonstrated higher probabilities of SY14-17 to be cost effectiveness compared to the other recovery assessment strategies for a WTP range between €30 and €160 (Fig. 4). This result was consistent with previous efficiency indicators and sensitivity analysis.

**Fig. 5:**
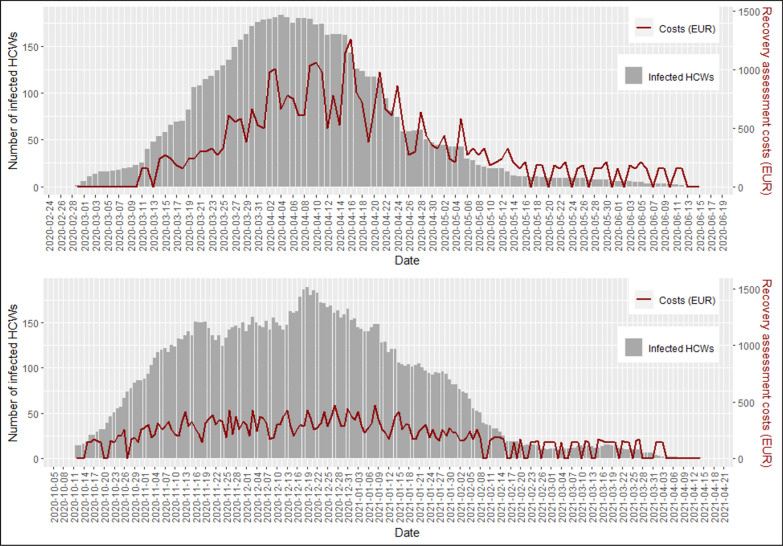
Daily number of infected health care workers (HCWs) and daily recovery assessment costs (human resources and microbiological infection diagnosis costs in local currency, euro—EUR) by date during the swab-based period (28/02/2020–12/10/2020; figure on top) and the symptoms-based period (from 13/10/2020; figure below

Daily recovery assessment costs were associated with the number of daily infected HCWs (p < 0.001), increasing of €28.3 (SE = 7.6e^−12^) as the number of daily infected individuals increase by one unit in both strategies (Fig. 5). As the number of infected HCWs increased, recovery assessment costs raised more in the swab-based strategy when compared with the standard symptoms-based (SY10-17) (β = 14.2 SE = 1.5e^−11^; p < 0.001).

## Discussion

The COVID-19 pandemic impacted the health care system of many countries at different levels. The cost in terms of patient’s health, human and financial resources was unprecedented. Fear of infection prevented people from seeking timely healthcare. This led to a 70% reduction in primary care visits [26]. The economic crisis resulting from COVID-19 pandemic and worldwide lockdown measures jeopardised the job of many people with a further burden for health. The 40% of respondents to a Commonwealth Fund survey declared they had health insurance through the job that was lost [27]. Moreover, HCWs were one of the most severely affected categories with a high risk of exposure to SARS-CoV-2 infection. This resulted in difficulties to ensure healthcare [28]. Median estimated recovery time was 21 days (Table 2). Similar results were shown by other authors with median time of viral clearance ranging from 17 to 24 days [29, 30]. This prolonged absence form work forced health facilities to reallocate human resources by prioritizing emergency and intensive care services. In a recent WHO survey of 39 European countries, it was found that the most affected sector was non-communicable diseases (NCDs) prevention and treatment. The disruption was consequent to a decline in inpatient volume from cancellation of elective care (reported by 75% of the countries), suspension of population screening services and closure of outpatient services [31]. In order to adequately plan the level of safe staffing, stakeholders and management should consider longer recovery time for those HCWs showing symptoms or being hospitalized. The severity of the disease was an independent risk factor for a longer recovery time and viral clearance [29, 32].

One of the greatest challenges posed by the COVID-19 pandemic has been finding the right balance between public health measures and restriction and the broader needs of society and the economy. In this perspective this study tries to explore the most cost-effectiveness recovery assessment strategy. Growing evidence showed that SARS-CoV-2 infected individuals are no longer infective after 21 days, except for specific categories such as the immunocompromised [33]. A symptoms-based strategy is therefore recommendable. Swab-based strategy proved to be dominated by the symptom-based one with an incremental cost of €2,508 and a negative incremental effectiveness when compared to the standard symptoms-based strategy with swab scheduled at 10 and 17 days. A symptoms-based strategy should be applied especially for those categories deemed essential such as HCW to shorten periods of isolation. When defining the infected HCWs management, an effective governance should aim to preserve adequate health assistance while safeguarding HCWs physical and mental health and preventing transmission to the patients and hospital staff [34].

When comparing different swabs scheduled for the symptoms-based strategy, the most cost-effective was the one with the swabs scheduled at 14 and 17 days. Similar results were obtained in a recent cost-effectiveness analysis of mass-testing screening in the general population [35]. At high transmission rates, the strategy with the best cost-effectiveness was the one with weekly swab test and a 14-days isolation period for the positive individuals. Interestingly, at low-moderate transmission rates, a 7-days isolation period was more cost-effective than either 10-day or 2-week periods. In the study sample, the symptoms-based strategy showed better performance as the number of infected HCWs increased compared to the swab-based strategy (Fig. 5). A shorter isolation period and recovery assessment with a single swab allowed faster reintroduction of HCWs to the workplace and avoided the addition of infected HCWs with increasing testing costs.

Currently, in Italy, high rates of vaccination and the spread of less severe variants have radically changed the epidemiological scenario. However, assessing the recovery of HCWs remains of primary importance, as for this particular population, according to Italian regulation, a negative swab test is still necessary to allow readmission at work. In addition, particular job categories such as healthcare workers deserve special attention. Returning to work prematurely after a SARS-CoV-2 infection could put the health of patients and colleagues at risk. For this reason, safe and effective yet cost-effective strategies to ensure recovery deserve to be investigated. The present results will allow to organize more cost-effective epidemic control strategies in the event of future pandemics.

This study has several limitations. First, the retrospective design and context-specificity of the included data may limit the generalizability of the results. Additionally, data on the vaccination status of HCWs were not considered because the vaccination campaign was in its beginning stages. However, this could have affected time and recovery probability. Data regarding the economic evaluation of nonpharmacological measures to contain COVID-19 pandemic are still scarce [36]. This implies uncertainty and a preliminary nature of many of the input parameters in this model. However, the PSA and OWSA were applied considering this uncertainty and giving robustness to the findings. In the cost effectiveness analysis were included only the directed costs related to the swab testing and the outcome was the number of working days saved for the infected HCWs. Future studies should try to explore the lost in Quality Adjusted Life Years (QALYs) of patients due to the reduction in the provision of health services related to the high burden of infected HCWs forced to stay on sick leave. This has a high level of complexity depending on the context and type of patient and disease. Moreover, many of the health and economic consequences of COVID-19 pandemics are not yet known.

## Conclusions

Since it has been proposed that we are in a transitional phase between pandemic and endemic, policymakers should maintain appropriate levels of vigilance. Especially for at-risk and strategically important groups such as HCWs, appropriate reintroduction measures after infection should be considered. In particular, the symptom-based strategy has been confirmed to be the most cost-effective and a 14- and 17-day swab schedule should be recommended. Moreover, the symptom-based strategy allowed the cost of recovery assessment to be contained as the rate of transmission and, consequently, the number of cases increased.

Few data exist on the evaluation of the economic impact of measures taken to counter the COVID-19 pandemic both at hospital level and broader levels. More insight should be given to these issues both for the present situation and for possible future similar scenarios.

## Supplementary Information

**Additional file 1. **Supplementary materials including additional data, graphic representation of the decision tree and the pseudocode of the algorithm used in the Monte Carlo simulation are provided.

## Data Availability

The datasets generated and/or analysed during the current study are available from the corresponding author on reasonable request.

## Abbreviations

CEA: Cost-effectiveness analysis
HSP: Health Surveillance Program
HCW: Healthcare workers
ICER: Incremental cost-effectiveness ratio
INMB: Incremental net monetary benefit
OWSA: One-way sensitivity analysis
PSA: Probabilistic sensitivity analysis
SY10-17: Standard symptoms-based strategies
SW: Swab-based
SY17: Symptoms-based strategies with swab scheduled at 17 days
SY14-17: Symptoms-based strategies with swabs scheduled at 14 and 17 days
UHV: University Hospital of Verona
WTP: Willingness to pay
WDS: Working days saved
WHO: World Health Organization
