# Supplementary material for: Comparing swab- and different symptoms-based strategies to ascertain COVID-19 recovery in healthcare workers: a cost-effectiveness analysis
Source: Cost Eff Resour Alloc. 2022 Sep 12;20:50. doi: 10.1186/s12962-022-00385-w (PMC9465662; doi:10.1186/s12962-022-00385-w)
Supplement: Supplementary file 1 — Additional file 1. Supplementary materials including additional data, graphic representation of the decision tree and the pseudocode of the algorithm used in the Monte Carlo simulation are provided. [file 12962_2022_385_MOESM1_ESM.docx]

**Additional file**


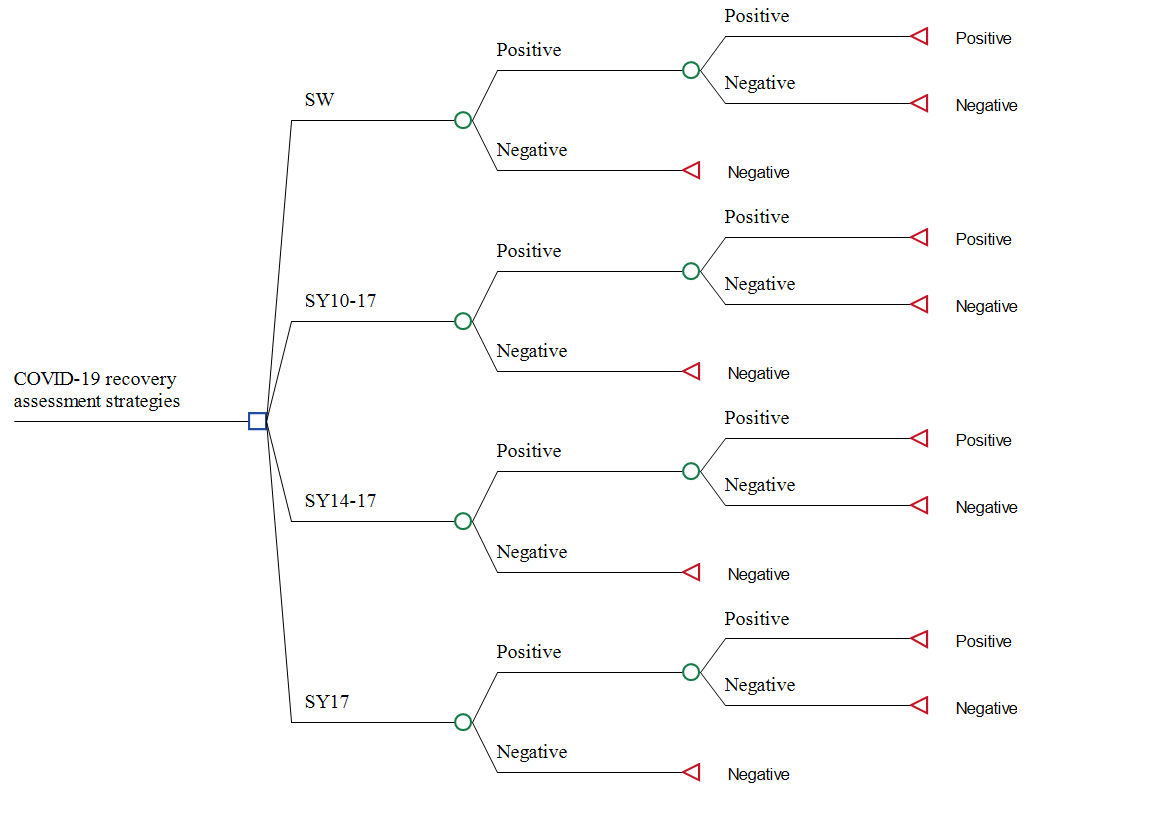


***t***

***t***

***t***

***t***

Figure S1 – Graphic representation of the decision tree used in Monte Carlo simulation. The knot denoted with “t” was repeated for every time point scheduled in the three different recovery assessment strategy. SW = Swab based strategy; SY10-17 = Symptoms 10-17 based strategy; SY14-17 = Symptoms 14-17 based strategy ; SY17 = Symptoms 17 based strategy.

Table S1 - Triangular distribution values (mode, minimum and maximum), used in Monte Carlo simulation, for recovery probability at every time point (t) scheduled by the four competing recovery assessment strategies according to the number of days since the first positive swab, estimated via Kaplan–Meier estimate.

|  | **SW strategy** | | | | **SY10-17 strategy** | | | | **SY14-17 strategy** | | | | **SY17 strategy** | | | |
| --- | --- | --- | --- | --- | --- | --- | --- | --- | --- | --- | --- | --- | --- | --- | --- | --- |
| **t** | **day** | **mode** | **min** | **max** | **day** | **mode** | **min** | **max** | **day** | **mode** | **min** | **max** | **day** | **mode** | **min** | **max** |
| 1 | 14 | 0.027 | 0.013 | 0.057 | 10 | 0.172 | 0.145 | 0.203 | 14 | 0.292 | 0.259 | 0.328 | 17 | 0.407 | 0.371 | 0.446 |
| 2 | 21 | 0.396 | 0.339 | 0.459 | 17 | 0.407 | 0.371 | 0.446 | 17 | 0.407 | 0.371 | 0.446 | 21 | 0.991 | 0.981 | 0.996 |
| 3 | 28 | 0.631 | 0.572 | 0.690 | 21 | 0.991 | 0.981 | 0.996 | 21 | 0.991 | 0.981 | 0.996 | 28 | 0.992 | 0.983 | 0.997 |
| 4 | 35 | 0.859 | 0.813 | 0.898 | 28 | 0.992 | 0.983 | 0.997 | 28 | 0.992 | 0.983 | 0.997 | 35 | 0.994 | 0.985 | 0.998 |
| 5 | 42 | 0.922 | 0.884 | 0.950 | 35 | 0.994 | 0.985 | 0.998 | 35 | 0.994 | 0.985 | 0.998 | 42 | 0.997 | 0.990 | 0.999 |
| 6 | 49 | 0.965 | 0.937 | 0.983 | 42 | 0.997 | 0.990 | 0.999 | 42 | 0.997 | 0.990 | 0.999 |  |  |  |  |

Table S2 – Frequencies of the job position of the SARS-CoV-2 infected healthcare workers distinguished by the two periods considered in the analysis

|  | First period (n=255) | Second period (n=661) | Overall (n=916) |
| --- | --- | --- | --- |
| **Job position** |  |  |  |
| Nurse | 265 (40,1%) | 92 (36,1%) | 357 (39,0%) |
| Other health professions* | 102 (15,4%) | 36 (14,1%) | 138 (15,1%) |
| Resident doctors | 86 (13,0%) | 47 (18,4%) | 133 (14,5%) |
| Senior doctors | 75 (11,3%) | 39 (15,3%) | 114 (12,4%) |
| Technical staff | 74 (11,2%) | 27 (10,6%) | 101 (11,0%) |
| Administrative staff | 52 (7,9%) | 14 (5,5%) | 66 (7,2%) |
| Scholarship | 7 (1,1%) | 0 (0,0%) | 7 (0,8%) |
| ********Healthcare assistant, Physiotherapist, Psychologist*  *First period from February 2020 to October 2020; Second period from October 2020 to April 2021* | | | |

**Pseudocode of Monte Carlo simulation algorithm**

**For** i = 1 to 10.000

**First step** (estimation of recovery probability values - π):

if strategy = SW OR SY10-17 OR SY14-17

for t = 1 to 6

π_strategy_(t)_ = random sample from triangular distribution of values in TableS1[strategy] at every time (t)

else

for t = 1 to 5

π_SY17_(t)_ = random sample from triangular distribution of values in TableS1[SY17] at every time (t)

**Second step** (estimation of healthcare workers recovery percentage - %π):

if strategy = SW OR SY10-17 OR SY14-17

%π_strategy_(1)_ = π_strategy_(1)_

for t = 2 to 6

% π_(t)__strategy = ((1- π_strategy_(t-1)_) * π_strategy_(t)_)

else

%π_SY17_(1)_ = π1_SY17_(1)_

for t = 2 to 5

%π_SY17_(t)_ = ((1- π_SY17_(t-1)_) * π_SY17_(t)_)

**Third step** (estimation of number of working days lost - WDL):

if strategy = SW OR SY10-17 OR SY14-17

for t = 1 to 6

WDL_strategy_(t) =_ random sample from normal distribution of column “day” in TableS1[strategy] at time (t)

else

for t = 1 to 5

WDL_SY17_(t) =_ random sample from normal distribution of column “day” in TableS1[SY17] at time (t)

**Fourth step** (estimation of costs):

cost = random sample from gamma distribution of costs in Table1

if strategy = SW

for t = 1 to 6

c_SW_(t)_ = cost*2 + c_SW_(t-1)_

if strategy = SY10-17 OR SY14-17

c_strategy_(1)_ = cost

for t = 2 to 3

c_strategy_(t)_ = cost *2

for t = 4 to 6

c_strategy_(t)_ = cost *3

else

for t = 1 to 2

c_SY17_(t)_ = cost

for t = 3 to 5

c_SY17_(t)_ = cost *2

**Fifth step** (estimation of effect - working days saved - WDS):

if strategy = SW OR SY10-17 OR SY14-17

for t = 1 to 6

WDS_strategy_(t)_ = 90 – WDL_strategy_(t)_

else

for t = 1 to 5

WDL_SY17_(t)_ = 90 – WDS_SY17_(t)_

**Sixth step** (estimation of total effect and cost for each strategy):

if strategy = SW OR SY10-17 OR SY14-17

effect_strategy = ∑^6^_t=1_ (%π_strategy_(t)_ * WDS_strategy_(t)_)

cost_strategy = ∑^6^_t=1_ (%π_strategy_(t)_ * c_strategy_(t)_)

else

effect_SY17 = ∑^5^_t=1_ (%π_SY17_(t)_ * WDS_SY_(t)_)

cost_SY17 = ∑^5^_t=1_ (%π_SY17_(t)_ * c_(t)__SY17)

**return** costs and effects for each strategy estimated at step six
